# Construction and applications of exon-trapping gene-targeting vectors with a novel strategy for negative selection

**DOI:** 10.1186/s13104-015-1241-6

**Published:** 2015-06-30

**Authors:** Shinta Saito, Kiyoe Ura, Miho Kodama, Noritaka Adachi

**Affiliations:** Graduate School of Nanobioscience, Yokohama City University, Yokohama, 236-0027 Japan; Graduate School of Science, Chiba University, Chiba, 263-8522 Japan; Advanced Medical Research Center, Yokohama City University, Yokohama, 236-0004 Japan

## Abstract

**Background:**

Targeted gene modification by homologous recombination provides a powerful tool for studying gene function in cells and animals. In higher eukaryotes, non-homologous integration of targeting vectors occurs several orders of magnitude more frequently than does targeted integration, making the gene-targeting technology highly inefficient. For this reason, negative-selection strategies have been employed to reduce the number of drug-resistant clones associated with non-homologous vector integration, particularly when artificial nucleases to introduce a DNA break at the target site are unavailable or undesirable. As such, an exon-trap strategy using a promoterless drug-resistance marker gene provides an effective way to counterselect non-homologous integrants. However, constructing exon-trapping targeting vectors has been a time-consuming and complicated process.

**Results:**

By virtue of highly efficient *att*-mediated recombination, we successfully developed a simple and rapid method to construct plasmid-based vectors that allow for exon-trapping gene targeting. These exon-trap vectors were useful in obtaining correctly targeted clones in mouse embryonic stem cells and human HT1080 cells. Most importantly, with the use of a conditionally cytotoxic gene, we further developed a novel strategy for negative selection, thereby enhancing the efficiency of counterselection for non-homologous integration of exon-trap vectors.

**Conclusions:**

Our methods will greatly facilitate exon-trapping gene-targeting technologies in mammalian cells, particularly when combined with the novel negative selection strategy.

**Electronic supplementary material:**

The online version of this article (doi:10.1186/s13104-015-1241-6) contains supplementary material, which is available to authorized users.

## Background

Gene targeting via homologous recombination provides a powerful means for studying gene function by a reverse genetic approach [1, 2]. This technology depends on homologous recombination reactions that occur between transfected DNA (i.e., targeting vector) and the host genome [3, 4]. In mammalian cells, however, this type of homologous recombination is quite a rare event. Even worse, targeting vectors integrate at overwhelmingly higher frequencies into random (or off-target) sites than into the target site [5], through non-homologous end-joining (NHEJ) [6] and NHEJ-independent mechanisms [7, 8]. For these reasons, in addition to positive selection using a drug-resistance marker gene (which is franked with two homology arms), strategies termed negative selection have been preferentially used in mammalian cell gene targeting to reduce the number of drug-resistant colonies associated with non-homologous vector integration into off-target sites [9].

To date, the most commonly used negative selection method is to place a counterselectable marker gene [e.g., a herpes simplex virus thymidine kinase gene or a gene coding for diphtheria toxin A fragment (*DT*-*A*)] outside of either (or both) of the homology arms. The presence of a toxic gene in the targeting vector is expected to kill random integrants (i.e., clones with off-target integration via non-homologous recombination), while having little impact on homologous recombination [10]. It appears, however, that this strategy has a limited efficacy of counterselection (2–3-fold enrichment at the most) and is not routinely applicable to gene-targeting experiments [11]. In fact, transient expression of *DT*-*A* is highly toxic to cells, resulting in a reduced colony formation rate after transfection. Another strategy to counterselect for non-homologous integration is to employ a promoterless drug-resistance gene as a positive-selection marker. In this case, the drug-resistance gene is only expressed functionally when fused, after vector integration into the host genome, to a coding sequence of expressed genes [12]. Although this strategy may not be applicable to genes with low expression levels, it has been reported that this type of targeting vectors is efficient in reducing the number of non-homologous integrants, without affecting absolute targeted integration frequencies [11]. More intriguingly, an exon-trap type of promoterless vectors has been shown to confer highly efficient gene targeting in mouse ES cells [13]. However, constructing such targeting vectors has been a time-consuming and complicated process, which typically involves the necessity to search for appropriate restriction sites for ligation reactions or vector linearization. Therefore, the success of vector construction depends crucially on the skills of researchers. Moreover, although exon-trap vectors are expected to provide an efficient tool for gene targeting, this technology has yet to be fully explored and still has room for improvement.

In this paper, we describe a simple and rapid method to construct exon-trap vectors that allow for efficient targeted gene disruption in mouse and human cells. Furthermore, we develop a novel method to enhance the efficiency of counterselection for non-homologous integration of exon-trap vectors. The combination of the rapid vector construction system and the novel negative selection strategy will greatly facilitate exon-trapping gene targeting in mammalian cells.

## Methods

### Vector construction

Oligonucleotides used to construct vectors are listed in Additional file 1: Table S1. All PCR reactions were performed with ExTaq^TM^ DNA polymerase (Takara Bio, Otsu, Japan) or KOD-Plus-DNA polymerase (Toyobo, Osaka, Japan). An entry clone plasmid, pENTR lox71-P, was constructed by inserting a 102-mer fragment containing *lox*71, *lox*P and several restriction sites into *Not*I-digested pENTR loxP plasmid [14]. The 102-mer DNA fragment was prepared by annealing oligonucleotides Lox71-P Fw and Lox71-P Rv (Additional file 1: Figure S1). The *lox*71 site is a mutant *lox*P site with 5 bp alterations [15].

To create entry clones for floxed promoterless markers, a hygromycin-resistance gene (*Hyg*^*R*^), a puromycin-resistance gene (*Puro*^*R*^), a neomycin-resistance gene (*Neo*^*R*^), and a bifunctional *lacZ*/*Neo*^*R*^ gene (*βgeo*) were each subcloned into pENTR lox71-P at the *Asc*I and/or *Cla*I sites, with an IRES, IRES2 or 2A peptide sequence added upstream of each drug-resistance gene. The IRES sequence was derived from encephalomyocarditis virus (ECMV) (Clontech, CA, USA; [16]). The IRES2 sequence is identical to the IRES sequence, except for the absence of 22-bp deletion at the 5′ side (Clontech; [17]). The 2A peptide sequence was derived from Thosea asigna virus (TaV) [18]. In this way, 12 entry clones (pENTR lox71P IRES-Hyg, pENTR lox71P IRES-Puro, pENTR lox71P IRES-Neo, pENTR lox71P IRES2-βgeo, pENTR lox71P IRES2-Hyg, pENTR lox71P IRES2-Puro, pENTR lox71P 2A-βgeo, pENTR lox71P 2A-Hyg, pENTR lox71P 2A-Puro, pENTR lox71P 2A-EGFP-2A-Puro, pENTR SA-IRES-Puro, and pENTR SA-IRES-Hyg) were constructed. Specifically, pENTR lox71P IRES-Hyg was constructed by subcloning a 2.2-kb *Xho*I/*Ngo*MIV fragment containing IRES, *Hyg*^*R*^ and polyA sequences into *Cla*I-digested pENTR lox71-P. pENTR lox71P IRES-Puro was constructed by subcloning a 1.3-kb *Mlu*I/*Pvu*I fragment containing IRES, *Puro*^*R*^ and polyA sequences into *Asc*I/*Cla*I-digested pENTR lox71-P. pENTR lox71P IRES-Neo was constructed by subcloning a 1.7-kb *Eco*RI/*Bam*HI fragment containing IRES, *Neo*^*R*^ and polyA sequences into *Cla*I-digested pENTR lox71-P. pENTR lox71P IRES2-βgeo was constructed with an In-Fusion^®^HD cloning kit (Clontech): a 4.7-kb fragment containing IRES2, *βgeo* and polyA sequences was PCR amplified with primers In-Fus Fw and In-Fus Rv, and the PCR fragment and *Cla*I-digested pENTR lox71-P were subjected to In-fusion cloning, yielding pENTR lox71P IRES2-βgeo. Likewise, pENTR lox71P IRES2-Hyg was constructed with In-Fusion^®^HD cloning kit (Clontech): a 1.9-kb fragment containing IRES2, *Hyg*^*R*^ and polyA sequences was PCR amplified with primers In-Fus Fw and In-Fus Rv, and the PCR fragment and *Cla*I-digested pENTR lox71-P were subjected to In-fusion cloning, yielding pENTR lox71P IRES2-Ηyg. pENTR lox71P IRES2-Puro was constructed by subcloning a 1.5-kb *Mlu*I/*Xho*I fragment containing IRES2, *Puro*^*R*^ and polyA sequences into *Asc*I/*Cla*I-digested pENTR lox71-P. pENTR lox71P 2A-βgeo was constructed by subcloning a 4.3-kb *Asc*I/*Acl*I fragment containing 2A peptide, *βgeo* and polyA sequences into *Asc*I/*Cla*I-digested pENTR lox71-P. A 4.3-kb fragment containing 2A peptide, *βgeo* and polyA sequences was PCR amplified with 2A-peptide sequence-containing primers (2AFw and 2ARv). pENTR lox71P 2A-Hyg was constructed by ligating a 1.1-kb *Bgl*II/*Not*I fragment containing *Hyg*^*R*^ and a 3.1-kb *Bam*HI/*Xba*I fragment containing 2A peptide and polyA sequences derived from pENTR lox71P 2A-βgeo. pENTR lox71P 2A-Puro was constructed by ligating a 0.6-kb *Bgl*II/*Eco*RV fragment containing *Puro*^*R*^ and a 3.1-kb *Bam*HI/*Xba*I fragment containing the 2A peptide and polyA sequences. pENTR 2A-EGFP-2A-Puro was constructed by ligating a 0.7-kb *Sma*I/*Sna*BI fragment containing 2A peptide and *EGFP* (Clontech; [19]) sequences and a 3.8-kb *Sma*I fragment containing 2A peptide, *Puro*^*R*^ and polyA sequences from pENTR lox71P 2A-Puro. pENTR SA-IRES-Puro was constructed by subcloning a 1.7-kb *Eco*RI/*Xho*I fragment containing SA (splice acceptor site; see below), IRES, *Puro*^*R*^ and polyA sequences into *Eco*RI/*Sma*I-digested pENTR loxP. pENTR SA-IRES-Hyg was constructed by subcloning a 2.6-kb *Eco*RV/*Pvu*II fragment containing SA, IRES, *Hyg*^*R*^ and polyA sequences into *Eco*RI/*Sma*I-digested pENTR loxP.

To generate targeting vectors for the human *HPRT* gene, 3.8 and 2.8-kb *HPRT* genomic fragments were PCR amplified with *att*B containing primers (HPRT-LH 5′Fw and HPRT 5′Rv for the 3.8-kb 5′ arm and HPRT 3′Fw and HPRT 3′Rv for the 2.8-kb 3′ arm). By using the MultiSite Gateway system (Life Technologies, Rockville, MD, USA), a floxed *Hyg*^*R*^ or *Puro*^*R*^ gene was placed between the 5′ and 3′ arms, thus yielding targeting vectors pHPRT-LH IRES2-Hyg and pHPRT-LH 2A-EGFP-2A-Puro. Similarly, another *HPRT* targeting vector, pHPRT-SH 2A-EGFP-2A-Puro, was constructed using the MultiSite Gateway system to assemble two homology arms (1.7 and 2.8-kb) and a drug-resistance gene cassette. Genomic fragments for homology arms were PCR amplified with *att*B containing primers (HPRT-SH 5′Fw and HPRT 5′Rv for the 5′ arm and HPRT 3′Fw and HPRT 3′Rv for the 3′ arm). All the plasmid vectors were purified with Qiagen Plasmid *Plus* Midi Kits (Qiagen K.K., Tokyo, Japan) and linearized with I-*Sce*I (New England Biolabs, Ipswich, MA, USA) prior to transfection [20].

The mouse *Rosa26* targeting vector, pmRosa26 IRES-Puro, was constructed using the MultiSite Gateway system. Briefly, 1.6 and 1.8-kb *Rosa26* genomic fragments were obtained by PCR using mouse genomic DNA as template and were used as 5′ and 3′ arms, respectively. The primers containing *att*B sequences, mRosa26 5′Fw and mRosa26 5′Rv were used for the 5′ arm and mRosa26 3′Fw and mRosa26 3′Rv for the 3′ arm. A *Puro*^*R*^ gene was inserted between the 5′ and 3′ arms to yield pmRosa26 IRES-Puro.

To construct a destination plasmid, pDEST SA–IRES–DTA–pA, the ORF of a gene encoding diphtheria toxin A fragment (*DT*-*A*) was PCR amplified with primers containing appropriate restriction sites for ligation reactions (DTA-Sal Fw and DTA-Not Rv) using pMC1DT-ApA plasmid (Kurabo, Osaka, Japan) as template. The PCR-amplified cDNA products were subcloned into pGEM^®^-T Easy Vector (Promega, Madison, WI, USA). Meanwhile, a splice acceptor site (SA; a 174-bp *Bam*HI fragment containing the adenovirus major late transcript splice acceptor sequence from the intron 1/exon 2 boundary) was excised from pSAβgeo [21]. The SA and *DT*-*A* fragments were sequentially inserted into the *Not*I and *Xho*I sites of pIRES (Clontech), respectively. Subsequently, a 1.8-kb *Bgl*II/*Pvu*I fragment containing the SA–IRES–*DT*-*A*–pA cassette was inserted into pDEST™R4-R3 (Life Technologies) at the *Afl*III site as illustrated in Additional file 1: Figure S2.

### Cell culture and transfection

The human fibrosarcoma cell line HT1080 was obtained from Institution for Fermentation (Osaka, Japan). HT1080 cells were maintained at 37°C in ES medium (Nissui Seiyaku, Tokyo, Japan) supplemented with 10% heat-inactivated calf serum (growth medium) in a humidified atmosphere of 5% CO_2_ in air. For passage, cells at late-log phase were washed once with Ca^2+^/Mg^2+^-free Phosphate-Buffered Saline (PBS^–^), and dispersed with 0.1% trypsin/PBS^−^ containing 0.02% EDTA for 5 min at 37°C; the cells were collected by low-speed centrifugation, resuspended in fresh growth medium at an appropriate density, replated onto 60- or 90-mm tissue culture dishes (NUNC, Roskilde, Denmark), and cultured.

Undifferentiated embryonic stem cells, CGR8 [22], were maintained on gelatin-coated dishes in the absence of feeder cells in DMEM (Nacalai Tesque, Kyoto, Japan) supplemented with 15% KSR (Life Technologies, Rockville, MD, USA), 1% fetal bovine serum (Thermo Fisher Scientific, Waltham, MA, USA), 0.1 mM β-mercaptoethanol (Wako Pure Chemical, Osaka, Japan), 1×MEM nonessential amino acids (Life Technologies) and LIF [22] at 37°C in a humidified atmosphere of 5% CO_2_ in air.

DNA transfection using the Nucleofector II system (Lonza, Basel, Switzerland) was performed according to the manufacturer’s instructions. Briefly, 2 × 10^6^ cells were suspended with 100 μl of the supplied solutions (Solution T), and transfected with 2 μg of linearized targeting vector. The cells were cultured for 48 h and then replated at a density of 2–4 × 10^5^ cells per 90-mm dish to determine the gene-targeting efficiency. Meanwhile, 1 × 10^2^ cells were replated onto 60-mm dish to determine the plating efficiency. After a 24-h incubation, hygromycin B (0.25–0.4 mg/ml, Wako Pure Chemical, Osaka, Japan) or puromycin (0.4 or 0.5 μg/ml, Wako Pure Chemical) was added to the sample plates. After a 10–14-day incubation, the resulting drug-resistant colonies were isolated and expanded to prepare genomic DNA for PCR and Southern blot analysis. DNA transfection using the MaxCyte STX device (MaxCyte, Gaithersburg, MD, USA) was performed according to the manufacturer’s instructions. Briefly, 4 × 10^7^ cells were suspended with 400 μl of the supplied solutions (MaxCyte^®^ Electroporation Buffer), and transfected with 40 μg of linearized targeting vector. Transfection into mouse ES cells was performed essentially as described previously [23].

### Gene targeting assay

The human *HPRT* targeting vector pHPRT-LH 2A-EGFP-2A-Puro or pHPRT-SH 2A-EGFP-2A-Puro was transfected into HT1080 cells, and puromycin-resistant colonies were counted to calculate the total integration frequency. Subsequently, single colonies were isolated and expanded to prepare genomic DNA. Gene-targeting events were screened by PCR analysis using primers HPRT-F and HPRT-R (Additional file 1: Table S1) and then confirmed by Southern blot analysis. The gene-targeting efficiency was calculated by dividing the number of targeted clones with that of puromycin-resistant clones analyzed. The targeted integration frequency was calculated by multiplying the total integration frequency by the targeting efficiency. The random integration frequency was calculated by subtracting the targeted integration frequency from the total integration frequency.

In mouse ES cells, gene-targeting assays were carried out essentially in the same manner as in HT1080 cells. Briefly, pmRosa26 IRES-Puro was linearized with I-*Sce*I and transfected into mouse ES cells. After a 2–3 week incubation, genomic DNA was prepared from puromycin-resistant clones and subjected to PCR analysis using primers mRosa26 5′ext and Universal primer C (Additional file 1: Table S1). The gene-targeting efficiency was calculated in the same manner as described above.

### Southern blot analysis

Southern blotting was performed as described previously [23]. Briefly, 30 μg of genomic DNA was digested with *Eco*RV and *Stu*I at 37°C and electrophoresed on a 0.8% agarose gel at 15 V for 14–18 h. Subsequently, DNA fragments were transferred to an Immobilon-Ny^+^ Transfer Membrane (Millipore, Concord Road, Billerica, MA, USA) by an alkaline transfer technique, followed by hybridization for >18 h at 55°C in hybridization buffer. The probe used was PCR amplified with primers HPRT 3′probe Fw and HPRT 3′probe Rv (Additional file 1: Table S1). Southern hybridization was performed using Amersham Gene Images AlkPhos Direct Labelling and Detection System (GE Healthcare Bio-Sciences, Piscataway, NJ, USA). Signals were detected with CDP-Star (Roche, Basel, Switzerland), and analyzed by using the Fuji Image Analyzer LAS-1000UVmini (Fuji Film Co., Tokyo, Japan).

## Results

### Rapid construction of exon-trapping targeting vectors using *att* site-mediated recombination

**Figure 1:**
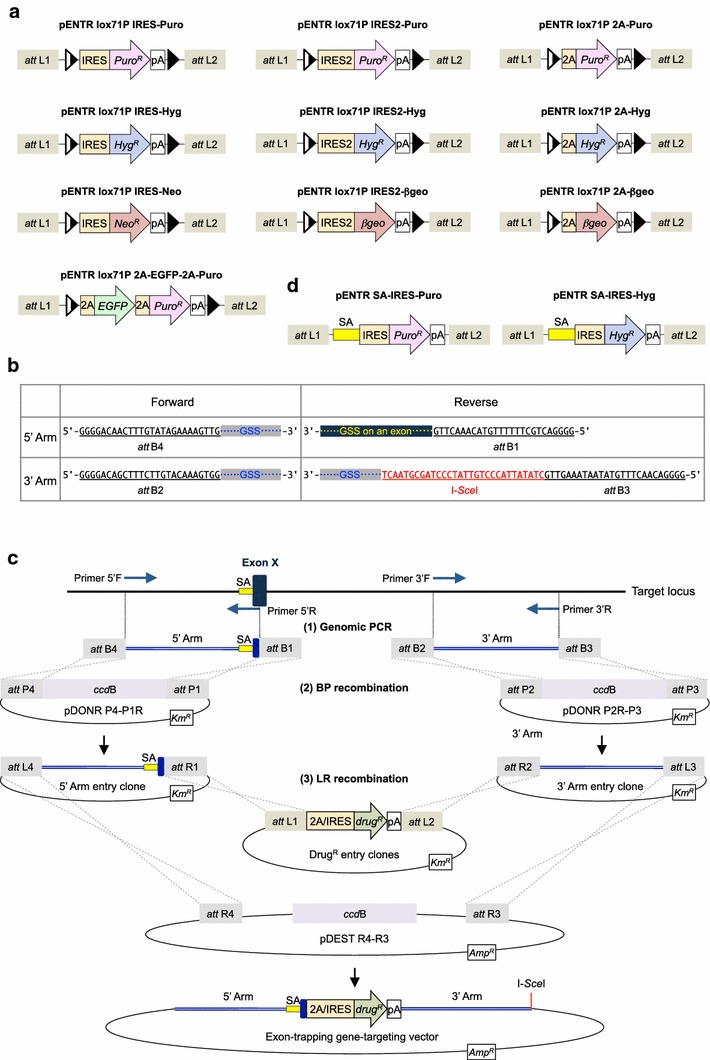
A simple and efficient method to rapidly construct exon-trapping targeting vectors. **a** Schematic representation of entry clones with floxed promoterless markers. For simplicity, the plasmid backbone is not drawn. *IRES* internal ribosome entry site, *2A* a 2A-peptide sequence derived from Thosea asigna virus (TaV), *Puro* ^*R*^ puromycin-resistance gene, *Hyg* ^*R*^ hygromycin-resistance gene, *Neo* ^*R*^ neomycin-resistance gene, *βgeo* *lacZ*/*Neo* ^*R*^, *EGFP* enhanced green fluorescent protein gene, *pA* polyadenylation signal. *Half-closed triangles* and *closed triangles* represent *lox*71 and *lox*P sequences, respectively. **b** Primer design for PCR amplification of homology arms. Each primer has four guanine residues at the 5′ end followed by an *att*B sequence. The four *att*B sequences *att*B4, *att*B1, *att*B2 and *att*B3 differ from one another, enabling efficient site-specific BP and LR recombination. The 5′-arm reverse primer should be set on the exon to be trapped (i.e., exon X in *panel* **c**), in order for the 5′-arm fragment to possess an authentic splice acceptor site at the 3′ side. The I-*Sce*I site added to the 3′-arm reverse primer facilitates linearization of the resulting targeting vector. *GSS* gene-specific sequences. *See text* for details. **c** Flow diagram of construction of targeting vectors based on the MultiSite Gateway system, which consists of three steps: (1) PCR amplification with *att*B-containing primers, (2) BP recombination between 5′ or 3′ arm fragment and a donor vector (pDONR P4-P1R or pDONR P2R-P3, respectively), and (3) LR recombination to yield the targeting vector by one-time assembly of four DNA fragments (*see text* for details). *SA* splice acceptor site, *drug* ^*R*^ drug-resistance gene, *Km* ^*R*^ kanamycin-resistance gene, *Amp* ^*R*^ ampicillin-resistance gene. **d** Schematic representation of pENTR SA-IRES-Puro and pENTR SA-IRES-Hyg. These two entry clones harbor an SA site-linked promoterless marker gene. See “Methods” for details.

To develop a system that enables rapid construction of exon-trapping targeting vectors, we employed the commercially available MultiSite Gateway^®^ Technology. This technology enables one-step assembly of four DNA fragments from different plasmids; namely, the backbone of a “destination vector” and three insert fragments subcloned in “entry clones”. Indeed, as we reported previously [14], the MultiSite Gateway system allowed us to rapidly construct conventional targeting vectors harboring a promoter-driven drug-resistance gene, which was originally subcloned in an entry clone plasmid. In this study, we first prepared a series of entry clones harboring a drug-resistance gene (*Puro*^*R*^, *Hyg*^*R*^, *Neo*^*R*^ or *βgeo*) flanked with *lox*71 and *lox*P sites (Figure 1a; Additional file 1: Figure S1). The use of floxed markers is of particular importance when one attempts to generate double- or triple-mutant cells, as a floxed region can be easily removed from the genome by transient expression of Cre recombinase in the cell. Importantly, the drug-resistance genes in these constructs do not possess a promoter; instead, an IRES or 2A peptide sequence is added upstream of each drug-resistance gene. Thus, these promoterless drug-resistance genes only acquire the ability to allow for positive selection when the ORF is fused to a coding sequence of expressed genes.

We next PCR amplified 5′ and 3′ homology arms by using four *att*B-containing primers. For these PCR reactions, the reverse primer for 5′-arm amplification should be set on an exon to be trapped (or disrupted), as depicted in Figure 1b. In this way, an authentic splice acceptor (SA) site is naturally incorporated at the 3′ side of the 5′-arm fragment. Also, the reverse primer for 3′-arm amplification contains an I-*Sce*I site (Figure 1b). This I-*Sce*I site permits linearization of the resultant targeting vector, without the need for extensive restriction mapping. The amplified fragments were then subjected to BP recombination to yield 5′ and 3′ arm-containing entry clones (Figure 1c). Finally, the three entry clones and the destination vector pDEST™R4-R3 were subjected to LR recombination to assemble the 5′ arm, an IRES/2A-linked drug-resistance gene, and the 3′ arm together, thus yielding the exon-trapping targeting vector (Figure 1c).

Using the method described above, we constructed several targeting vectors for the mouse *Rosa26* gene and the human *HPRT* gene (Additional file 1: Figure S3). Importantly, we were able to construct these vectors shorter than 10 days, usually within 1 week. In addition, the two subcloning steps using BP and LR recombination were quite efficient, with little or no appearance of *E. coli* transformants harboring incorrect plasmids.

In general, designing a primer on an exonic sequence is easy and beneficial in performing an efficient and highly specific PCR reaction, given that exonic sequences tend to be more unique than are intronic sequences, which are rich in repetitive DNA sequences. In some cases, however, one may favor to set the 5′-arm reverse primer (i.e., the 3′ end of the 5′ arm) on an intronic sequence. We therefore constructed additional entry clones, in which a drug-resistance gene cassette possesses an SA site upstream of the IRES sequence (Figure 1d; pENTR SA-IRES-Puro and pENTR SA-IRES-Hyg). These entry clones will be useful for constructing a targeting vector that is designed to trap an intronic region of the target gene.

### Exon-trapping gene targeting in mouse ES cells and human HT1080 cells

To evaluate the usefulness of exon-trap vectors for actual gene-targeting experiments, we examined whether the *Rosa26* targeting vector described above was competent for gene disruption in mouse ES cells. Electroporation of I-*Sce*I-linearized pmRosa26 IRES-Puro into mouse ES cells (1.3 × 10^7^ cells) gave rise to 13 puromycin-resistant clones, and 2 clones were found to be correctly targeted. Thus, the targeting efficiency was considerably high (15.4%), even though the targeting vector contained relatively short homology arms (1.6 and 1.8 kb). We next employed a human cell line, HT1080, to evaluate *HPRT* targeting vectors. The HT1080 cell line has a near-diploid karyotype [24] and has been frequently used for studies on gene targeting [25–27]. Using a MaxCyte electroporation system, I-*Sce*I-linearized pHPRT-SH 2A-EGFP-2A-Puro was transfected into HT1080 cells. As shown in Table 1, the *HPRT* gene was successfully knocked out in these cells.

**Table 1:** Exon-trap-based *HPRT*-gene targeting in HT1080 cells

Experiment	Number of cells transfected	Total number of puromycin-resistant colonies	Gene-targeting efficiency
1	4.0 × 10^7^	411	0.8% (2/244)
2	4.0 × 10^7^	614	0.4% (2/501)

The targeting vector pHPRT-SH 2A-EGFP-2A-Puro was I-*Sce*I linearized and transfected into HT1080 cells, and the gene-targeting efficiency was determined as described in “Methods”.

### ExTraPANS: a novel negative selection cassette that efficiently counterselects for non-homologous integration into off-target genomic sites

**Figure 2:**
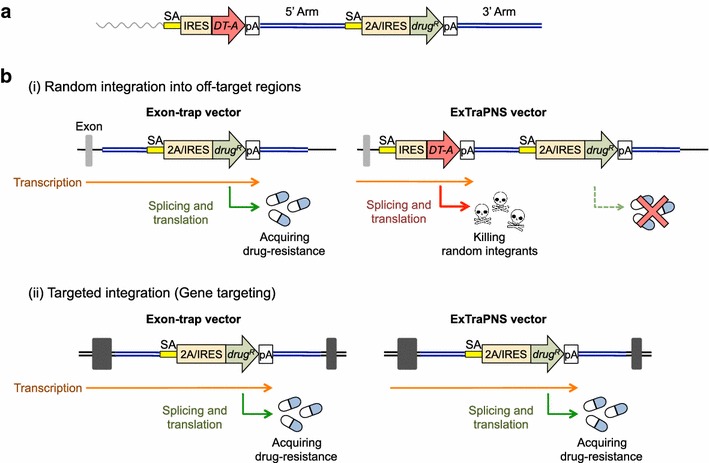
Schematic representation of the novel negative selection strategy. **a** Schematic representation of a conditionally cytotoxic gene cassette composed of an SA site, an IRES sequence, a *DT*-*A* gene and a polyA sequence. This cassette, when placed upstream of the 5′ arm of an exon-trapping targeting vector, is expected to function as a negative selection marker. **b** Schematic representation of the impact of the negative selection cassette on integration events. When an exon-trap vector integrates non-homologously into a gene-coding region, the cells are capable of acquiring drug resistance (*i*, *left panel*). In contrast, when an exon-trap vector possesses the negative selection cassette (ExTraPANS vector), the upstream SA site would trap the splicing from the upstream exon (*grey box*) to allow *DT*-*A* gene expression, thereby killing random integrants (*i*, *right panel*). On the other hand, the presence of the negative selection cassette is expected not to affect homologous recombination-mediated targeted integration (*ii*). Abbreviations are as in Figure 1.

In the experiments described above, exon-trap vectors were successfully utilized to disrupt mammalian genes in a desired manner. In human somatic cells, however, the targeting efficiency was not as high as expected, owing to the occurrence of a large number of drug-resistant colonies associated with non-homologous integration of exon-trap vectors into off-target sites. We therefore sought to examine whether exon-trap-based gene targeting could be further enhanced when combined with an additional strategy. For this aim, we designed a novel negative selection cassette composed of a promoterless toxic gene, as shown in Figure 2a. In this cassette, the *DT*-*A* gene does not have a promoter and, therefore, is not expressed in a transient manner; instead, an SA site and an IRES sequence are added upstream of the *DT*-*A* gene in a manner similar to promoterless drug-resistance genes for positive selection. We anticipated that when this cassette is placed upstream of the 5′ arm of an exon-trapping targeting vector, non-homologous integrants that are otherwise capable of acquiring drug resistance would express the *DT*-*A* gene and thus fail to form viable colonies, as depicted in Figure 2b. In contrast, targeted integration via homologous recombination would not be affected.

We next inserted this cassette (i.e., SA–IRES–*DT*-*A*–polyA sequence) into the destination vector pDEST™R4-R3 (near the *att*R4 site), and named the resulting plasmid pDEST SA–IRES–DTA–pA (Additional file 1: Figure S2). We then constructed targeting vectors for the mouse *Rosa26* and human *HPRT* genes in the same manner as described above, except that pDEST SA–IRES–DTA–pA was employed instead of pDEST™R4-R3. When these targeting vectors were transfected into HT1080 cells, a significant reduction in the number of drug-resistant clones was observed. As shown in Table 2, the absolute integration frequency was decreased ~5–25-fold when the negative selection cassette was present in the targeting vector. More importantly, by virtue of reduced integration frequencies, the targeting efficiency was actually enhanced when the targeting vector possessed the negative selection cassette (Table 3; Experiments 1–3). We further employed mouse ES cells and found that the targeting efficiency at the *Rosa26* locus was enhanced by the presence of the negative selection cassette in the targeting vector (Table 3; Experiment 4). These results demonstrate that the novel negative selection cassette with a conditionally cytotoxic *DT*-*A* gene is indeed effective in further reducing the number of non-homologous off-target integrants. We termed this novel strategy “ExTraPANS”, which stands for *ex*on-*tra*pping *p*ositive *a*nd *n*egative *s*election.

**Table 2:** Effect of the novel negative selection cassette on integration frequency

Experiment	*DT*-*A*	Random integration frequency^a^	Fold decrease
1	−	1.0 × 10^−4^ (1)	25
	+	4.1 × 10^−6^ (0.04)	
2	−	2.8 × 10^−5^ (1)	8.5
	+	3.3 × 10^−6^ (0.12)	
3	−	6.2 × 10^−5^ (1)	5.6
	+	1.1 × 10^−5^ (0.18)	

The targeting vector pHPRT-SH 2A-EGFP-2A-Puro was I-*Sce*I linearized and transfected into HT1080 cells by using the Nucleofector II system (Experiments 1 and 2) or the MaxCyte devise (Experiment 3). *DT*-*A* denotes the presence (+) or absence (−) of the ExTraPANS cassette (SA–IRES–*DT*-*A*–polyA sequence) in the targeting vector. The random-integration frequency was determined as described in “Methods”.

^a^Numbers in parentheses represent relative random-integration frequencies.

**Table 3:** Effect of the novel negative selection cassette on targeting efficiency

Experiment	Selection marker	*DT*-*A*	Gene-targeting efficiency	Fold increase
1	2A-EGFP-2A-Puro	−	0.4% (1/250)	9.0
		+	3.6% (2/55)	
2	2A-Puro	−	1.7% (2/116)	2.8
		+	4.7% (2/43)	
3	IRES2-Hyg	−	0% (0/133)	>4.6
		+	3.7% (1/27)	
4	IRES-Puro	−	0% (0/7)	>3.5
		+	50% (1/2)	

Human HT1080 cells and mouse ES cells were used for targeted disruption of the *HPRT* locus (Experiments 1–3) or the *Rosa26* locus (Experiment 4), respectively. *DT*-*A* denotes the presence (+) or absence (−) of the ExTraPANS cassette (SA–IRES–*DT*-*A*–polyA sequence) in the targeting vector.

## Discussion

In this paper, we have established a simple and rapid method to construct exon-trap vectors that enable to disrupt virtually any locus of expressed genes of interest in a desired manner. Our vector construction system is simpler and quicker than any other methods reported thus far. Furthermore, we have developed a novel strategy for negative selection to enhance gene targeting by virtue of dramatically reduced random-integration frequencies. Since the negative selection cassette (SA–IRES–*DT*-*A*–polyA) has been placed in a ready-to-use destination vector (pDEST SA–IRES–DTA–pA), rapid construction of exon-trap vectors harboring this cassette can be easily performed using the MultiSite Gateway^®^ Technology, usually within 1 week. Thus, our strategy described here is usefully applicable to gene-knockout/knock-in experiments in human somatic cells as well as mouse ES cells.

In somatic cell gene-knockout experiments, multiple marker gene cassettes are needed for the purpose of disrupting more than one locus [14]. Thus, we have constructed a series of floxed promoterless marker genes that allow for positive selection with puromycin, hygromycin, or neomycin (G418). Indeed, we have confirmed that all of these cassettes are competent as a positive selection marker in human somatic cells (our unpublished observations). Previous work has suggested that although an exon-trap type of targeting vectors is useful for mammalian cell gene targeting, the efficiency likely depends on cellular expression levels of the target gene [21]. For this reason, we employed a 2A peptide sequence as well as an IRES (or IRES2) sequence for each of the selection markers. The IRES/IRES2 sequences allow expression of multiple ORFs from the same mRNA, with weaker expression of a downstream gene(s). In contrast, 2A peptide sequences allow expression of multiple genes on a single ORF at the same level [28]. We therefore expected that 2A peptide-linked drug-resistance genes might be three-times more effective in reducing random integrants than IRES-linked markers, because the 2A peptide sequence must be fused in-frame to a coding sequence of expressed non-target genes in order for cells to acquire drug resistance. However, a significant number of random integrants were actually observed with a 2A-linked drug-resistance gene, while we were able to obtain correctly targeted clones with satisfactory efficiencies. We speculate that 2A peptide sequences could have more chance than IRES sequences to confer drug resistance when non-homologously integrated into genes with low expression levels.

Accumulating evidence indicates that the targeting efficiency is stimulated by a DNA break introduced at the chromosomal target site [29–31]. In particular, recently developed methods using artificial nucleases such as ZFN, TALEN or CRISPR have been dramatically improving genome-engineering technologies [32–34]. It is worth noting, however, that such nuclease-triggered gene targeting is capable of causing mutations owing to unexpected off-target effects [35–37]. For this reason, using artificial nucleases may not be desirable or should be avoided in some cases. We therefore refocused on simpler strategies that could be combined with exon-trapping targeting vectors, and have succeeded in developing a novel strategy termed ExTraPANS that can even more efficiently counterselect for random integration events. With the use of the ExTraPANS strategy, we have shown that the targeting efficiency can be elevated to as high as ~4.7% in human HT1080 cells. Yet, there is still room for improvement. In fact, although we had expected that random-integration frequencies might drop to zero with the ExTraPANS strategy, we actually encountered an unignorable number of random integrants. Two possibilities may explain the imperfect efficacy of our current strategy. One possibility is that, prior to or during the non-homologous integration event, the targeting vector has been nucleolytically degraded to lose (at least part of) the negative-selection cassette sequence, thereby disabling *DT*-*A* expression. In support of this view, frequent terminal deletions have been observed with chromosomally integrated plasmid vectors in mouse ES cells [38] and in human somatic cells (our unpublished observations). Another possibility is that, even when the negative selection cassette remained intact after integration, the SA sequence we employed could not work efficiently at certain loci and thus *DT*-*A* was not fully expressed to exert cytotoxicity. Such functional inertness of ectopic SA sites in the genome has been reported earlier in experiments using exon-trap vectors [39]. Since our preliminary experiments suggest that these two possibilities both seem likely, we are currently trying to overcome these issues to further enhance the efficacy of the ExTraPANS strategy.

## Conclusions

In summary, we have established an efficient method to perform exon-trap-based gene targeting in mammalian cells. The novel strategy using a promoterless *DT*-*A* gene has the capacity to significantly reduce the number of off-target integrants. Although the efficiency of exon-trapping gene targeting may largely be influenced by the expression level of the target gene, the usefulness of the ExTraPANS strategy is quite intriguing and must be emphasized. Our methods described here will greatly facilitate gene-knockout/knock-in experiments in mammalian cells.

## Additional file

Additional file 1
**Table S1**. Oligonucleotides used in this study. **Figure S1**. Construction of pENTR lox71-P. **Figure S2**. Construction of pDEST SA-IRES-DTA-pA. **Figure S3**. Schematic representation of exon-trapping gene targeting at the mouse Rosa26 and human HPRT loci.

## References

[1] Capecchi MR (1989). Altering the genome by homologous recombination. Science.

[2] Vasquez KM, Marburger K, Intody Z, Wilson JH (2001). Manipulating the mammalian genome by homologous recombination. Proc Natl Acad Sci USA.

[3] Langston LD, Symington LS (2004). Gene targeting in yeast is initiated by two independent strand invasions. Proc Natl Acad Sci USA.

[4] Kan Y, Ruis B, Lin S, Hendrickson EA (2014). The mechanism of gene targeting in human somatic cells. PLoS Genet.

[5] Yanez RJ, Porter AC (1998). Therapeutic gene targeting. Gene Ther.

[6] Lieber MR (2010). The mechanism of double-strand DNA break repair by the nonhomologous DNA end-joining pathway. Annu Rev Biochem.

[7] Iiizumi S, Kurosawa A, So S, Ishii Y, Chikaraishi Y, Ishii A (2008). Impact of non-homologous end-joining deficiency on random and targeted DNA integration: implications for gene targeting. Nucleic Acids Res.

[8] Ishii A, Kurosawa A, Saito S, Adachi N (2014). Analysis of the role of homology arms in gene-targeting vectors in human cells. PLoS One.

[9] Mansour SL, Thomas KR, Capecchi MR (1988). Disruption of the proto-oncogene int-2 in mouse embryo-derived stem cells: a general strategy for targeting mutations to non-selectable genes. Nature.

[10] Yagi T, Nada S, Watanabe N, Tamemoto H, Kohmura N, Ikawa Y (1993). A novel negative selection for homologous recombinants using diphtheria toxin A fragment gene. Anal Biochem.

[11] Hanson KD, Sedivy JM (1995). Analysis of biological selections for high-efficiency gene targeting. Mol Cell Biol.

[12] Stanford WL, Cohn JB, Cordes SP (2001). Gene-trap mutagenesis: past, present and beyond. Nat Rev Genet.

[13] Friedel RH, Plump A, Lu X, Spilker K, Jolicoeur C, Wong K (2005). Gene targeting using a promoterless gene trap vector (“targeted trapping”) is an efficient method to mutate a large fraction of genes. Proc Natl Acad Sci USA.

[14] Iiizumi S, Nomura Y, So S, Uegaki K, Aoki K, Shibahara K (2006). Simple one-week method to construct gene-targeting vectors: application to production of human knockout cell lines. Biotechniques.

[15] Albert H, Dale EC, Lee E, Ow DW (1995). Site-specific integration of DNA into wild-type and mutant lox sites placed in the plant genome. Plant J.

[16] Rees S, Coote J, Stables J, Goodson S, Harris S, Lee MG (1996) Bicistronic vector for the creation of stable mammalian cell lines that predisposes all antibiotic-resistant cells to express recombinant protein. Biotechniques 20(1):102–104, 106, 108–110 10.2144/96201st05 8770413

[17] Jang SK, Krausslich HG, Nicklin MJ, Duke GM, Palmenberg AC, Wimmer E (1988). A segment of the 5′ nontranslated region of encephalomyocarditis virus RNA directs internal entry of ribosomes during in vitro translation. J Virol.

[18] Szymczak AL, Vignali DA (2005). Development of 2A peptide-based strategies in the design of multicistronic vectors. Expert Opin Biol Ther.

[19] Cormack BP, Valdivia RH, Falkow S (1996) FACS-optimized mutants of the green fluorescent protein (GFP). Gene 173(1 Spec No):33–38 10.1016/0378-1119(95)00685-0 8707053

[20] Adachi N, Kurosawa A, Koyama H (2008). Highly proficient gene targeting by homologous recombination in the human pre-B cell line Nalm-6. Methods Mol Biol.

[21] Friedrich G, Soriano P (1991). Promoter traps in embryonic stem cells: a genetic screen to identify and mutate developmental genes in mice. Genes Dev.

[22] Niwa H, Masui S, Chambers I, Smith AG, Miyazaki J (2002). Phenotypic complementation establishes requirements for specific POU domain and generic transactivation function of Oct-3/4 in embryonic stem cells. Mol Cell Biol.

[23] So S, Adachi N, Lieber MR, Koyama H (2004). Genetic interactions between BLM and DNA ligase IV in human cells. J Biol Chem.

[24] Rasheed S, Nelson-Rees WA, Toth EM, Arnstein P, Gardner MB (1974). Characterization of a newly derived human sarcoma cell line (HT-1080). Cancer.

[25] So S, Nomura Y, Adachi N, Kobayashi Y, Hori T, Kurihara Y (2006). Enhanced gene targeting efficiency by siRNA that silences the expression of the Bloom syndrome gene in human cells. Genes Cells.

[26] Itzhaki JE, Porter AC (1991). Targeted disruption of a human interferon-inducible gene detected by secretion of human growth hormone. Nucleic Acids Res.

[27] Yanez RJ, Porter AC (2002). A chromosomal position effect on gene targeting in human cells. Nucleic Acids Res.

[28] Szymczak AL, Workman CJ, Wang Y, Vignali KM, Dilioglou S, Vanin EF (2004). Correction of multi-gene deficiency in vivo using a single ‘self-cleaving’ 2A peptide-based retroviral vector. Nat Biotechnol.

[29] Rouet P, Smih F, Jasin M (1994). Expression of a site-specific endonuclease stimulates homologous recombination in mammalian cells. Proc Natl Acad Sci USA.

[30] Paques F, Duchateau P (2007). Meganucleases and DNA double-strand break-induced recombination: perspectives for gene therapy. Curr Gene Ther.

[31] Grizot S, Smith J, Daboussi F, Prieto J, Redondo P, Merino N (2009). Efficient targeting of a SCID gene by an engineered single-chain homing endonuclease. Nucleic Acids Res.

[32] Miller JC, Holmes MC, Wang J, Guschin DY, Lee YL, Rupniewski I (2007). An improved zinc-finger nuclease architecture for highly specific genome editing. Nat Biotechnol.

[33] Miller JC, Tan S, Qiao G, Barlow KA, Wang J, Xia DF (2011). A TALE nuclease architecture for efficient genome editing. Nat Biotechnol.

[34] Ran FA, Hsu PD, Wright J, Agarwala V, Scott DA, Zhang F (2013). Genome engineering using the CRISPR-Cas9 system. Nat Protoc.

[35] Petek LM, Russell DW, Miller DG (2010). Frequent endonuclease cleavage at off-target locations in vivo. Mol Ther.

[36] Fu Y, Foden JA, Khayter C, Maeder ML, Reyon D, Joung JK (2013). High-frequency off-target mutagenesis induced by CRISPR-Cas nucleases in human cells. Nat Biotechnol.

[37] Hsu PD, Scott DA, Weinstein JA, Ran FA, Konermann S, Agarwala V (2013). DNA targeting specificity of RNA-guided Cas9 nucleases. Nat Biotechnol.

[38] Suzuki K, Ohbayashi F, Nikaido I, Okuda A, Takaki H, Okazaki Y (2010). Integration of exogenous DNA into mouse embryonic stem cell chromosomes shows preference into genes and frequent modification at junctions. Chromosome Res.

[39] Skarnes WC (2000). Gene trapping methods for the identification and functional analysis of cell surface proteins in mice. Methods Enzymol.

