# Supplementary material for: Construction and applications of exon-trapping gene-targeting vectors with a novel strategy for negative selection
Source: BMC Res Notes. 2015 Jun 30;8:278. doi: 10.1186/s13104-015-1241-6 (PMC4486125; doi:10.1186/s13104-015-1241-6)
Supplement: Additional file 1 — Table S1. Oligonucleotides used in this study. Figure S1. Construction of pENTR lox71-P. Figure S2. Construction of pDEST SA-IRES-DTA-pA. Figure S3. Schematic representation of exon-trapping gene targeting at the mouse Rosa26 and human HPRT loci. [file 13104_2015_1241_MOESM1_ESM.pdf]

Construction and applications of exon-trapping gene-targeting vectors with a novel strategy for negative selection

Shinta Saito, Kiyoe Ura, Miho Kodama, and Noritaka Adachi

SUPPLEMENTARY INFORMATION

Table S1. Oligonucleotides used in this study.

Figure S1. Construction of pENTR lox71-P. An entry clone plasmid, pENTR lox71-P, was constructed by inserting a 102-mer fragment containing *lox*P, *lox*71 and several restriction sites into *Not*I-digested pENTR loxP (Iiizumi, S., *et al*. (2006) *BioTechniques*, **41**, 311-316). The *lox*71 site is a mutant *lox*P site with 5 bp alterations (shown in red). Abbreviations are as in Figure 1.

Figure S2. Construction of pDEST SA-IRES-DTA-pA. To construct the pDEST SA-IRES-DTA-pA plasmid, the ORF of a gene encoding diphtheria toxin A fragment (*DT-A*) was PCR amplified with primers containing appropriate restriction sites for ligation reactions (DTA-Sal Fw and DTA-Not Rv; Supplementary Table S1) using pMC1DT-ApA plasmid (Kurabo, Osaka, Japan) as template. The PCR-amplified cDNA products were subcloned into pGEM®-T Easy Vector (Promega, Madison, WI, USA). A 0.7-kb *Not*I fragment containing the *DT-A* ORF was subsequently subcloned into *Not*I-digested pIRES (Clontech). Meanwhile, a 174-bp *Bam*HI fragment containing the adenovirus major late transcript splice acceptor sequence from the intron 1/exon 2 boundary was excised from pSAbgeo (Friedrich, G. and Soriano, P. (1991) *Genes Dev.*, **5**, 1513-1523), and subcloned into *Xho*I-digested pIRES DTA plasmid. Finally, a 1.8-kb *Bgl*II/*Pvu*I fragment containing the SA–IRES–*DT-A*–pA cassette was inserted into pDEST™R4-R3 (Life Technologies, Rockville, MD, USA) at the *Afl*III site, thus yielding pDEST SA-IRES-DTA-pA. Abbreviations are as in Figure 1.

Figure S3. Schematic representation of exon-trapping gene targeting at the mouse *Rosa26* and human *HPRT* loci. (**A**) The *Rosa26* locus, the *Rosa26* targeting vector, and the targeted locus are shown. Black boxes, half-closed triangles and closed triangles represent exons, *lox*71 and *lox*P sequences, respectively. Red arrows indicate PCR primers (see Supplementary Table S1). (**B**) The *HPRT* locus, the targeting vectors (pHPRT-SH 2A-EGFP-2A-Puro, pHPRT-LH 2A-EGFP-2A-Puro and pHPRT-LH IRES2-Hyg), and the disrupted loci are shown.

Supplementary Table S1. Oligonucleotides used in this study

Name	Sequence
Lox71-P Fw	5'-GGCCGCTACCGTTCGTATAGCATACATTATACGAAGTTATGGCGCGCCATCGATGCGATCGCATAACTTCGTATAGCATACATTATACGAAGTTATAA-3'
Lox71-P Rv	5'-GGCCTTATAACTTCGTATAATGTATGCTATACGAAGTTATGCGATCGCATCGATGGCGCGCCATAACTTCGTATAATGTATGCTATACGAACGGTAGC-3'
DTA-Sal Fw	5'-GTCGACATGGATCCTGATGATGTTGTTGAT-3'
DTA-Not Rv	5'-GCGGCCGCTTAGAGCTTTAAATCTCTGTAGGTA-3'
In-Fus Fw	5'-TATGGCGCGCCATCGGGTTAATTAACAGCTGACGCGTGC-3'
In-Fus Rv	5'-TATGCGATCGCATCGCGTATTACCGCCTTTGAGTGAGCT-3'
2A Fw	5'-GGCGCGCCCGGGGAGGGCAGAGGAAGTCTTCTAACATGCGGTGACGTGGAGGAGAATCCCGGCCCTATGGATCCCATGGCGGATCCCGTCGTTTTAC-3'
2A Rv	5'-AACGTTACGTATTACCGCCTTTGAGTGAGCTG-3'
HPRT-LH 5’ Fw	5'-GGGGACAACTTTGTATAGAAAAGTTGCACATCACAGGTACCATATCAGTG-3'
HPRT-SH 5’ Rv	5'-GGGGACAACTTTGTATAGAAAAGTTGCAGCAGCTGTTCTGAGTACTTGCT-3'
HPRT 5’ Rv	5'-GGGGACTGCTTTTTTGTACAAACTTGCACATCTCGAGCAAGACGTTCAGT-3'
HPRT 3’ Fw	5'-GGGGACAGCTTTCTTGTACAAAGTGGCCTGCAGGATCACATTGTAGCCCTCTGTGTGC-3'
HPRT 3’ Rv	5'-GGGGACAACTTTGTATAATAAAGTTGCTATATTACCCTGTTATCCCTAGCGTAACTCAGGGTAGAAATGCTACTTCAGGC-3'
HPRT−F	5'-TGAGGGCAAAGGATGTGTTACGTG-3'
HPRT−R	5'-TTGATGTAATCCAGCAGGTCAGCA-3'
HPRT 3’ probe Fw	5'-GCAAGCTACATAGTACTAAGCCAC-3'
HPRT 3’ probe Rv	5'-TGGCCTATAGCCACCATGGAAGCT-3'
mRosa26 5’ Fw	5'-GGGGACAACTTTGTATAGAAAAGTTGTTGGGACATGGATTTCTCCGGTG-3'
mRosa26 5’ Rv	5'-GGGGACTGCTTTTTTGTACAAACTTGATCTCGAAGACCTGTTGCTGCTC-3'
mRosa26 3’ Fw	5'-GGGGACAGCTTTCTTGTACAAAGTGGTGTTCATAATCCCAAGATGTTGCC-3'
mRosa26 3’ Rv	5'-GGGGACAACTTTGTATAATAAAGTTGCTATATTACCCTGTTATCCCTAGCGTAACTCCACTTAAGACACCAGTTTCAGCC-3'
mRosa26 5’ ext	5'-CCTAATGAGCCACTATGGATGTGG-3'
Universal primer C	5'-TCCAAGCGGCTTCGGCCAGTAACG-3’

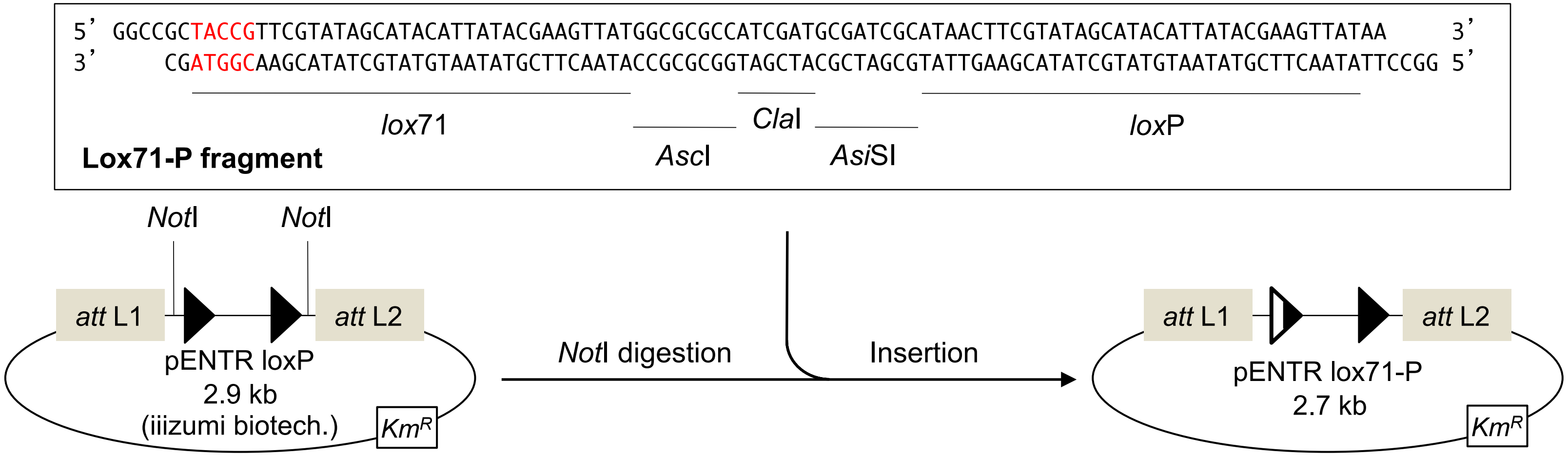

Supplementary Figure S1

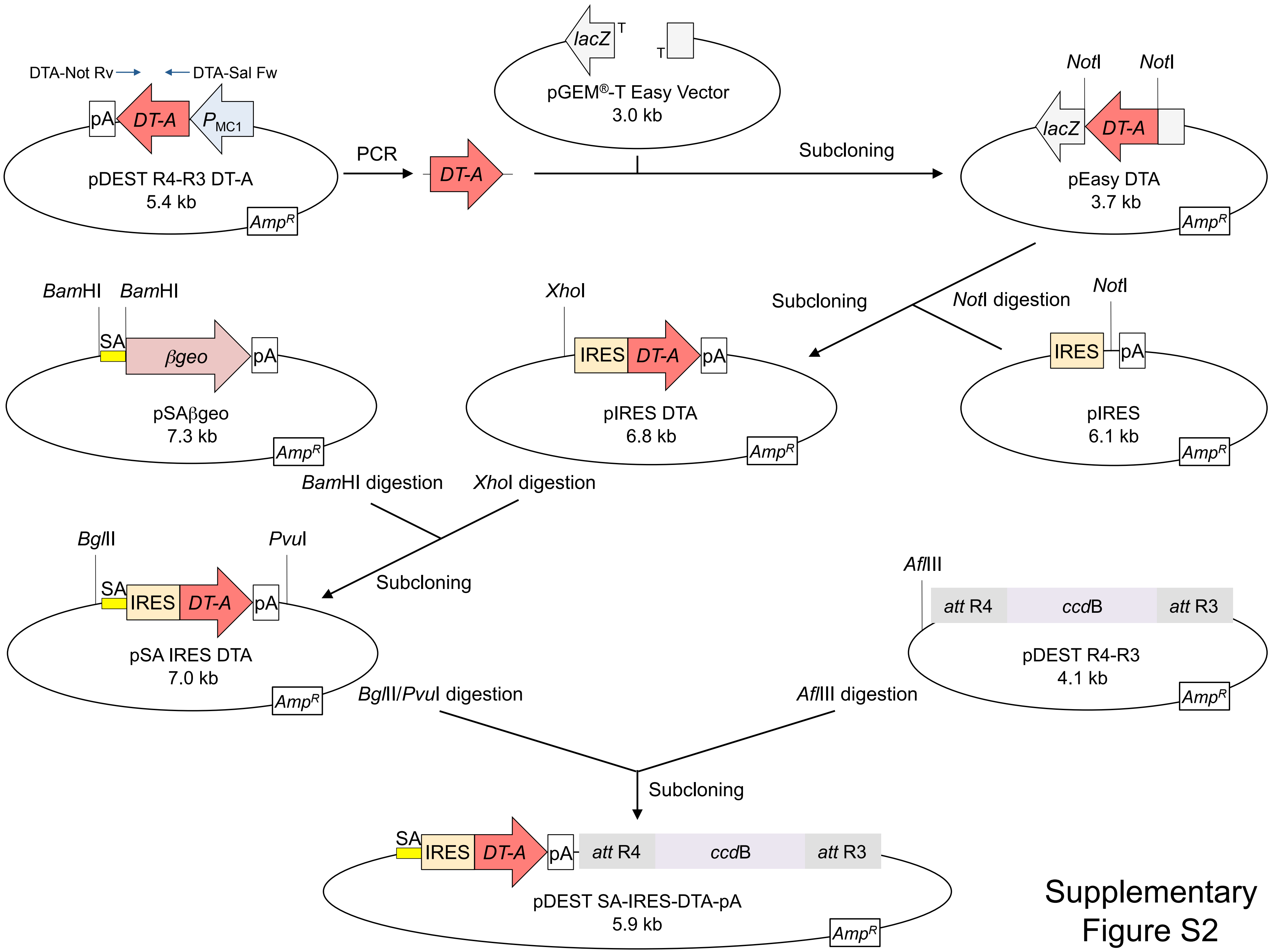

Supplementary Figure S2

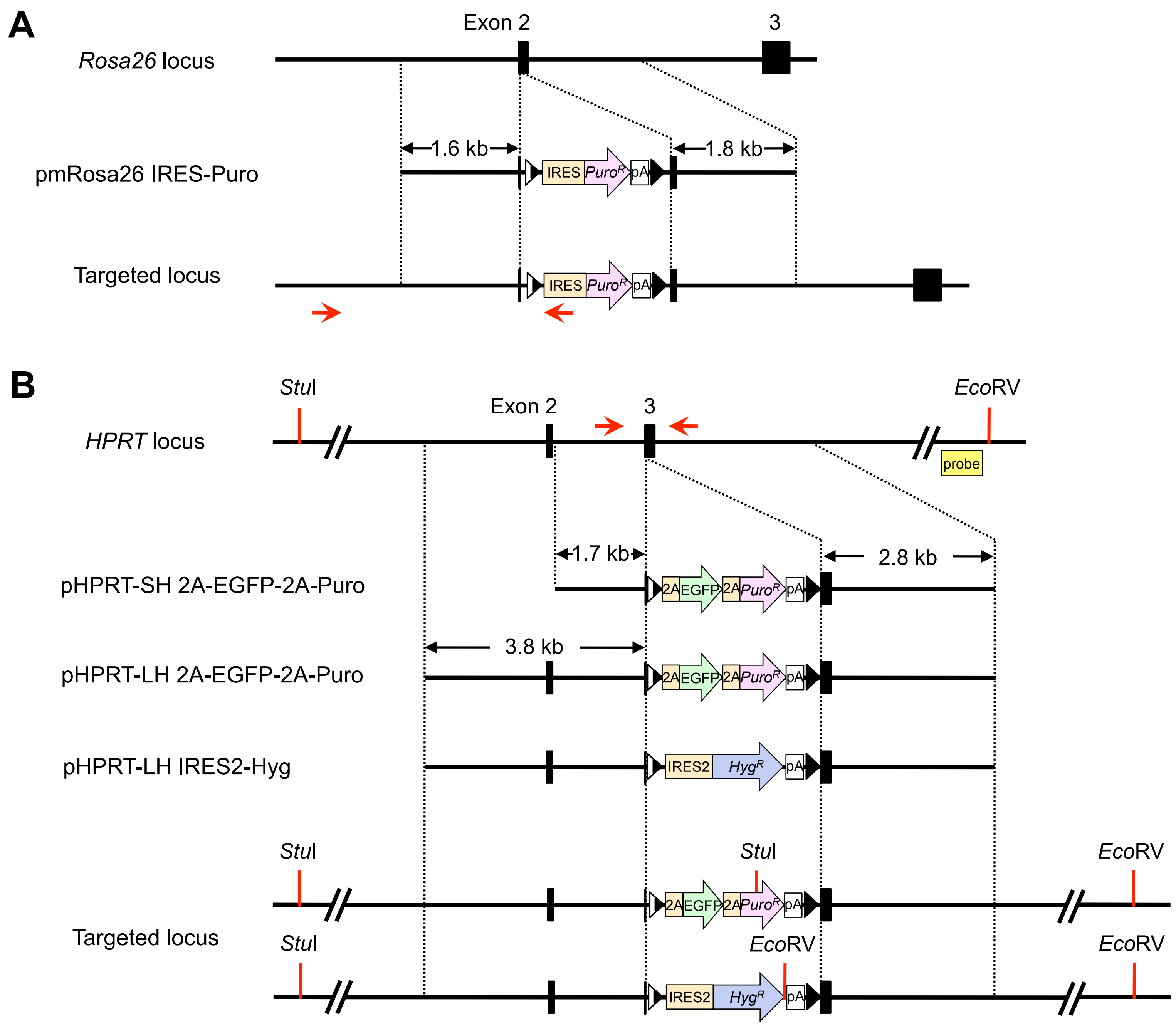

Supplementary Figure S3
